# Use of external fixators in developing countries: a short socioeconomic analysis

**DOI:** 10.1186/s12962-022-00353-4

**Published:** 2022-03-29

**Authors:** Pathmanathan Cinthuja, P. C. I. Wijesinghe, Pujitha Silva

**Affiliations:** 1 Department of Allied Health Sciences, Faculty of Medicine, University of Colombo, Colombo, 00800 Sri Lanka; 2 Colombo North Teaching Hospital, Ragama, 11010 Sri Lanka; 3 Department of Electronic and Telecommunication Engineering, Center for Biomedical Innovation (CEBI), Biomedical Engineering, University of Moratuwa, Moratuwa, 10400 Sri Lanka

**Keywords:** Low cost, External fixators, Developing countries, Socioeconomic

## Abstract

The use of external fixators (EFs) dates back to 377 BC Hippocrates’ time, and it has a wide range of orthopaedic applications. External fixator has expanded its use in the management of fractures and other musculoskeletal conditions. It is widely used all over the world to manage complex musculoskeletal injuries. It has many advantages as compared to internal fixation in some trauma scenarios. However, the cost of the external fixators presents a dilemma to the healthcare system in developing countries. The goals of this review article are to explain the importance of EFs in developing countries in managing fractures, to determine the problems encountered at present during external fixation by developing countries, to identify solutions that could be used to address these issues, expand the use of external fixation into other domains of treatment, the impact of COVID-19 pandemic on fracture management based on existing literature. In conclusion, EFs are very expensive, researches have been conducted to overcome these barriers in developing countries. However, there are limitations in implementing in developing countries. It is important to have affordable and clinically acceptable EFs available in developing countries.

## Introduction

A higher percentage of severe fractures occurs in the developing world. The estimated percentage of severe fractures is about 80% Gellman [1]. The level of development measured by per capita gross national income is used to classify countries. According to that, high-income (more than $12,375), upper-middle-income ($3996 and $12,375), lower-middle-income ($1026 and $3995) and low income (less than $1025) are the categorie [2]. According to the United Nations categorisation the World Bank low and middle-income countries are considered “developing” countries [3]. In developing countries, urbanisation and motorcycles are common causes for high energy trauma [4]. High energy fracture is defined as falling from a height higher than ≥ 10 feet and motor vehicle accidents [5, 6] Heavy industry, construction, and transportation are factors associated with urbanisation that cause high energy trauma [7]. Road traffic injuries are the third-largest contributor to the global burden of disease [1]. The main reasons for a higher number of road traffic accidents are poorly maintained road systems, overcrowding, and the number of people travelling in each vehicle [1]. Gun violence is another reason for fracture in developing countries [8]. The common causes of fractures among children in developing countries are poor local laws, poor adult supervision of young children, poor quality and lack of well-designed public recreational and sports facilities [9]. In developing countries, natural disasters are another reason for fractures [10, 11].

External Fixators (EFs) have been used for over 2000 years to immobilise fractures while preserving soft tissue integrity [12] The first use of a true EF device dates back to 377 BC during Hippocrates time, and it has been used to treat closed tibial fractures [13]. It was made up of leather rings connected by four wooden rods from a cornel tree that covered the limb [13]. In 1840 Malgaigne introduced the concept of external skeletal fixation [14]. In 1897, the first readily available external fixator was introduced [14]. External fixation is the use of pins and/ or thin wires implanted percutaneously in bone, connected outside the body by clamps, rods, and other metal or composite devices; it is used to stabilise fractures and reconstruct complex orthopaedic deformities [15]. It is used for temporary fixation (of fractures), definitive correction of limb length discrepancies and congenital malformations [16]. The use of EFs provides an opportunity to improve the quality of treatment [4]. Gustillo Grade III fractures, grade II fractures, infected fractures, pseudoarthrosis, and corrective osteotomies are the classical indications for the use of external fixation devices [17]. The principal goals of using EFs are to achieve rapid fracture stability for vascular intervention, wound debridement, and as part of damage control orthopaedics in polytrauma patients and achieving bone stability without disruption of the resuscitation process [18]. Ease of wound care, quick mobilisation of the patient, and shortening hospital stay are advantages of external fixators’ treatment [17]. Uniplanar, biplanar, multiplanar, unilateral, bilateral, circular fixator [12], and Hybrid are sub categorisations of EFs. Common complications associated with external fixators include pin-site infection, pin loosening [19]. Other less common complications include neurovascular injury, mechanical failure, septic joint, pin tract osteomyelitis, and pin over-penetration [20].

Increasing incidence and complexity of fractures, limited health care facilities such as inadequate operating theatre facilities, lack of equipment, lack of expertise, long distances to a healthcare facility and patient or family ignorance are barriers to effective management of fracture in developing countries [21]. Over 50% of the world’s traumatic injuries occur in low and middle-income countries [8]. External fixation techniques are widely utilised for fracture stabilisation in developing countries due to increasing trauma accidents [8].

Poor infrastructure and hygiene conditions of the infrastructure are some of the reasons for choosing external fixation beyond its true indications [4]. These include lack of availability of operation theatre time, long waiting list in public hospitals, unavailability of definitive fixation devices, unavailability image intensifier, lack of intensive care beds for post-operative care [22], prolong anaesthesia time and poor sterility in theaters [23]. The high costs of commercially available devices (including EFs) present a dilemma [4] as it places burden on healthcare costs of developing countries [4]. EFs are reused most of the time considering the cost associated with external fixation frame components [24, 25].

Many studies have introduced low-cost EFs to overcome high-cost issues associated with EFs [4, 8, 26–28]. Also, some studies have been conducted to analyse the socioeconomic impact of EFs [29, 30].

This review article aims to explain the importance of EF in developing countries in managing fractures, to determine the problems encountered in using EFs in developing countries, to identify solutions that could be given to address these issues, to expand the use of external fixation into other domains of treatment, the impact of COVID-19 on fracture management and need for local manufacturing of EFs.

### Review process outline

A literature search was performed in PubMed and Google scholar. Studies published up to 2021 were included. Studies published in English and studies related to EFs in developing countries were included. Sample keywords included are EFs, developing countries, complications, fracture fixation reuse, socioeconomic, other domains, and deformity corrections.

### The importance of EFs in developing countries in managing (open) fractures

In many developing countries, fractures are managed by the application of plaster-of-Paris cast after limited wound debridement. This is associated with complications such as chronic osteomyelitis, joint stiffness, sepsis, non-union, and malunion. It can be managed, and complications associated with Plaster-of-Paris can be minimised by using various EFs. However, there are many limitations to accessing EFs in developing countries [3, 31].

The use of EFs avoids extensive soft tissue damage and facilitates the management of associated soft tissue injuries [16, 32]. It is considered suitable for underdeveloped and developing countries as it provides good results for open fractures (of the tibia) [33–35]. EFs are a good choice in developing countries as it does not require specialised facilities or theatres. Therefore it is considered suitable for developing countries [33].

An EF is considered advantageous among the high-risk geriatric population as it minimise problems associated with prolonged recumbency [36, 37], avoidance of delay, shorter duration of surgery, minimal blood loss, and shorter hospital stay. It is considered an acceptable alternative in this population considering co-morbidities and limited resources in developing countries [38].

EFs are widely used in developing countries as there are many incidences of deformities due to neglected trauma and birth defects (e.g., clubfoot deformity, pediatric hip disorders, congenital limb length discrepancies) compared to developed countries. According to a study conducted in 2015 in Haiti, it showed that 99% of the success rate was observed in deformity correction using (Taylor Spatial Frame) EFs [39].

Many developing countries are adversely affected due to war [40] High-energy weapons or blast injuries usually result in extensive tissue damage. More than 75% of all injuries in wars are localised to the extremities, and more than 1/3 of those injuries are accompanied by bone fractures [32]. External fixation is considered a definitive choice of treatment of war-related open fractures of extremities and produces good late functional results [41]. EFs are used in low-technology environments of certain war zones [25].

According to a retrospective study conducted in Israel, staged external fixation protocol was used to treat 64 high-energy limb fractures caused by gunshots and blasts. The study results indicated that a staged EF is a valuable strategy for treating war injuries to extremities [32]. The use of EFs can have some financial benefits for poor countries in a war where their economies are already burdened.

### The problems encountered at present during external fixation by developing countries

Prioritising funds for infectious diseases are commonly seen in low- and middle-income countries [1]. Also, there is an increasing burden of non-communicable diseases across all countries [42]^.^ Non-communicable diseases are given priority in the sustainable development agenda in developing countries [43]. Therefore it is likely to neglect fund allocations to trauma services. In many developing countries, lower limb fracture management is done by prolonged traction, long leg cast splinting and immobilisation. In contrast, in developed countries, lower limb fractures are treated with early surgical intervention (based on restoring anatomy and stability as soon as possible). Faster recovery, early mobilisation, superior long-term functional outcomes are advantages of early surgical correction [1].

Incidence of pin tract infections, loss of fixation, loosening of the components [44], wound infection, deep infection, loss of reduction, delayed union [45], Iatrogenic vascular injuries [46], stiff joint, nerve injury, pain, and swelling [47], cellulitis [48] are complications associated with EFs reported in various studies.

EFs are used as temporary frames, which are used for a limited period before the definitive fixation of skeletal injuries [29]. They are not designed to withstand a physiological load. EFs are reused most of the time considering the cost associated with external fixation frame components [24]^.^ Device response to the initial application, device response to initial mechanical break-in period, mechanical wear properties and fatigue during extended use, reprocessing control, liability for device failure, and fiduciary consideration are some reasons for precluding reuse of EFs [15, 49]. However, some studies showed a potential for saving up to 25% by reusing EF frames [50], up to 34% saving of mean cost for a fixator [44].

It is unknown how many times a fixator can be reused. A study was conducted in India using locally manufactured EFs; the results mentioned that rods and clamps were reused 10 times, and screws on average 4 times. The reuse of EFs reduced the cost per patient from the US $ 50 to the US $12 [25]. It is indicated in a study that EFs are used up to three times in a developed country [50]; there was no difference in the rate of reoperation or complications [50]. Another study suggested that an EF (rod) can be reused at least once [51], it was tested using Hoffmann II EF, 8-mm carbon fibre rods were used for the Hoffmann II EF [51], it showed a 6% difference in bending stiffness among new and reprocessed. Another study tested static mechanical testing on EFs after one or two clinical uses; it indicated no catastrophic mechanical failures [52]. These results indicate that the EFs can be used multiple times, although there is no clear indication of the limit on the number of times of re-use.

Non-profit hospitals use reprocessed EFs compared to for-profit hospitals as it is cost-effective [53]. Litigation is considered a significant barrier for EF reprocessing systems [53]. Some studies indicate no significant difference between new and recycled EFs in terms of pin tract infection, loss of fixation, and loosening of components [24, 50].

Studies have been conducted to analyse the socioeconomic impact of EFs. The contributing factors are the total cost of the external fixation frame [29], the total cost of medical care, and the total absence from work or school [30].

Another factor to be considered is availability of human resources. It is reported in studies that poor human resource allocation in the (peripheral) hospitals in developing countries [11, 54–58]. According to a report from Sri Lanka in 2005, only 22 consultants orthopedics surgeons were working in the government sector managing a population of 18 million. Further it was reported that in peripheral hospital, usually 1 orthopedic surgeon and 2 general surgeons were managing 2 million people. Initial management was done by the general surgeon on call, it was reported that orthopedic experience of general surgeon is variable [11]. Another study from Ghana reported that there are 24 orthopedic surgeons in Ghana, it is 0.10 orthopedic surgeons per 100,000 people. The study results suggested that specialists are in short supply in developing countries and “Brain drain” is mentioned as a primary cause [55]. A report from Malawi in 2005 mentioned that only 4 orthopedic surgeons to care a population of 12 million people [59].

### Solutions that could be used to address these issues

The high costs of commercially available devices present a dilemma to healthcare [4]. High cost and trauma complexity are reasons for the failure of proper external fixation treatment [8]. The high cost of EFs puts a burden on healthcare costs of developing countries [4]. Various studies have been conducted to find possible solutions to overcome the problems associated with EFs in developing countries.

In India, a study was conducted to compare the cost-effectiveness of managing tibia fractures using external and internal fixators. According to the study results, it was concluded that locally made EFs of open tibial fractures are cost-effective in rural India [25].

EFs are simple frames applied externally to the body and are not invasive. There is a price difference between manufacturing products locally and importing from other countries; it is applicable for EFs too. It may be important to note that the high price is placed on some of the imported EFs. According to a circular issued by Sri Lankan Inland Revenue Department in 2016, it is mentioned that the tax is exempted for importing raw materials for the production or manufacture of medical purpose products [60]. Therefore, if EFs, or some components of EFs, are produced locally, these can be very affordable. However, there are some exceptions for an invasive component of the fixator. The Schnz pins are an example as they need a level of precision in fabrication and quality, being the invasive component of the fixator.

Many studies have introduced a low-cost EF to overcome high-cost issues associated with EFs [4]. A low-cost EF should be rigid enough to facilitate fracture healing without secondary loss of reduction [4]. An EF should have the following characteristics: simplicity, the versatility of application, the ability to minimise soft-tissue damage, stability at the bone-screw interface, rigidity, and cost-effectiveness [4].

A study was conducted to overcome limitations due to high-cost EFs. A novel, inexpensive, unilateral fixator was constructed using 3D printed clamps and readily available supporting components. According to the study, 3D printed EFs meet the need for low-cost EFs in developing countries. However, efficacy and safety studies should be conducted before being deployed in clinical settings [8].

Manufacturing a typical fixator for a more affordable cost using a different choice of material to make the fixator is another possible option. It has been investigated for many years. In 1988, an inexpensive EF was designed using galvanised iron pipe and mild steel bolts and nuts, manufactured in a hospital workshop with a minimum of tools [26] (Ali Noor, 1988). Goh et al. analysed AG (Alinoor-Goh), a new simple and low-cost EF [27], against commercially available EFs (AO). In 2001, ring fixators’ rings were manufactured from tubes cast from scrap aluminium; it was recommended for surgical treatment [28]. There was a 20% to 25% cost reduction compared to conventional EFs when EFs are manufactured using low-cost plastic resin [61]. Kousassi et al. analysed the 304L stainless steel EF to manage simple and comminuted patterns [4].

Cost for EFs of lower extremity was studied. $ 5252 (± $1798) was the average wholesale cost of an EF construct for tibial plafond fracture. It was noted that the clamp was the major contributor to cost for each construct [62]. The study reported cost of different components such cost of self-drilling schanz screw 80 mm thread/200 mm is $253.00, Large EF 11 mm carbon fiber rod 300 mm/ mr-conditional is $348.00, 6.0 mm transfixation pin 225 mm is $193.00, large EF pin clamp mr-conditional/ 6-position is $673.00 large EF combination clamp mr-conditional is $946.00, large EF multi-pin clamp mr-conditional/ 4-position Component cost is $1013.00 [62]. This study concluded that small changes in the construct design can significantly impact the cost. Another study analysed the cost of EFs for pelvic and lower extremity injuries. $5900 was noted as the average cost per external fixation frame [29]. According to a study conducted in India, the cost of a newly assembled EF is US $50; this included locally manufactured rods, clamps and standard implant-grade 316L steel screws [23].

A cost analysis is done for low cost EFs manufactured in Sri Lanka and the United Kingdom. According to the manufacturing cost analysis, a linear EF costs around £110/set (Airfreight charges of £24.50/set in addition to the manufacturing cost) in UK, Stryker Hoffman fixator costs around £1000/set (manufactured in India) and Stryker Hoffman fixator costs around £2700/set (manufactured in the UK).

In addition to manufacturing cost, the cost of treatment should be considered in the cost analysis of EFs. This can be divided into two categories, direct and indirect costs. Direct costs include treatment, hospitalization, and outpatient appointments. Loss of productivity can be considered as an indirect cost. Further, cost analysis should include the cost related due to complications [40]. Complications can cause a longer hospital stay, increased cost of care for patients, and increased loss of working hours [40, 63]. This might have serious economic problems as in many developing countries there are no insurance policies available for everyone.

A study conducted in Tanzania analysed the costs related to external fixation for open diaphyseal fractures of tibia. The mean total cost per patient for EF was $559 ± 70.5. The mean operation time 74.6 ± 5.1 min and the mean hospital stay was 2.44 ± 1.47 days for the EF group. The theatre cost was 76% of the total cost ($425), hospital per diem costs were 17.5% ($ 97), follow-up costs were around 4.5% ($26) and investigation costs were around 2% ($11.2). The cost was relatively high compared to patients managed with Intramedullary nail ($ 425.8 ± 38) due to repeated hospital visits or readmission for pin tract infections and nursing care [32, 64].

A study conducted in Pakistan among adults with diaphyseal fracture of tibia indicates patients managed with EF had a shorter hospital stay, low rate of infection, high rate of early union compared to patients managed with Plaster of Paris [31, 41].

The mean duration of hospital stay in developing countries was analysed. According to a study from Nigeria, the mean duration of stay for patients with tibail fracture was 8.6 ± 6.3 weeks and the femoral fractures group was 20.0 ± 6.3 weeks [40]. An audit conducted in Sri Lanka on open tibia fracture management in a selected hospital indicates that patients were managed with EF, internal fixation or Plaster of Paris after initial debridement and the median duration for exchange external fixation or POP to tibia nails was 8.5 ± 4 days. The study mentioned that the median duration of hospital stay of the patients after definitive surgery or plastic intervention was 16 ± 5 days [42, 65]. Results of a study from Tanzania indicate mean length of hospital stay for patients with tibial fractures managed with EFs was 2.44 ± 1.47 days [32, 64]. A study from a developed country indicates that the average mean duration of overall hospital stay was 7.8 days [43] for tibial plateau fractures managed with EF. These results show that there is a variation in the length of hospital stay and it depends on several factors.

Appropriate education of healthcare providers is one of the major barriers in developing countries [54]. Training for orthopedic surgeons in developing countries can be categorised into three broad categories such as carry out training within their country, carry out in the same region and carry out in more developed countries. In many developing countries, the third option is the widely used training method. In Sri Lanka, MD in Orthopedic surgery training programme is conducted in two stages. Those are basic orthopedic training and higher orthopedic training which consists of one year of advanced level training in two different orthopedic units in a teaching hospital. After successful completion of MD examination, there will be a further 2 years of training in Orthopedics and Trauma, one year will be spent overseas and one year in Sri Lanka [66].

Training overseas has both advantages such as training usually of a high standard, exposure to modern technology, establishing academic and professional links and friendships, and disadvantages such as differences in pathology in more developed countries, to that of the developing world countries [59]. Complications related to orthopedic trauma are different in developing countries compared to developed countries. Most often, books written by authors from developed countries are referred to, they might not cover the common problems encountered in developing countries [54]. Therefore, it is indicated in a report that there should be a balance in the curriculum between developing countries’ needs and recent advances [54]. It is reported in a study that the academic mission of service, training and research should be an essential component of orthopedic surgical training programmes [67].

### Use of external fixation into other domains of treatment: deformity correction and managing bone loss

EFs have been used in several other treatment domains such as in reconstructive orthopaedics and fracture care. EFs are used for arthrodesis [68], treatment of failing or failed femoral plating [69], infected non-union fracture (femoral shaft) [70], treatment of bone loss [71, 72], deformity correction [73–75] and treatment of osteomyelitis [76].

In developing countries, more than 220,000 children are born with clubfoot every year [77]. The Ponseti method is a gold standard treatment for clubfoot. It is an affordable method as it uses casting materials [77]. There are many children with neglected clubfoot and disabilities due to wrongly treated or untreated clubfoot. Ponseti principles are used to treat many neglected clubfeet [78]. However, surgical approaches are more likely beneficial for older children and adults with neglected clubfeet [69]. A study was conducted among patients with severe and recurrent congenital talipes equinovarus deformities, it showed that complete correction was obtained using (Ilizarov) EFs and had relatively few complications [79]. An EF is a safe and economically feasible procedure for reconstructive surgery of Charcot feet, and it improves activity level, minimises complications, and reduces secondary amputation [68, 75].

Studies have been conducted to evaluate the corrective capability of EFs in the treatment of neglected clubfoot. The results revealed that EFs allow correction of clubfoot deformities [73, 75, 80]. There is reducing risks of cutaneous or neurovascular complications and avoiding excessive shortening of the foot [81] and a high rate of excellent results, with low frequency of complications [82].

Deformity correction is one of the domains where an EF is widely used. Chronic haemophilic arthropathy with fixed flexion deformity was corrected using a (circular) EF; the study results showed that EF is an important, safe, and less invasive alternative surgical treatment modality with a low recurrence rate [83].

An EF is used for limb lengthening surgery. According to a study conducted in Hong Kong, lengthening up to 40% of the initial length of the bone segment was achieved without significant long-term sequelae using EFs. Either Ilizarov or Orthofix was used [84].

Surgical techniques, materials used in EFs, the introduction of hexapod style fixators, innovative configuration and pin modifications are advances made in the EFs. Similar to changing the material, having a different configuration of external fixator is also beneficial as it maximizes the construct stability [85]. Different external fixator configurations are possible for the management of a deformity [85]. Selecting the most suitable assembly shape is an important and fundamental step before performing correction of deformity [86]. Introducing new external fixators to correct multiplane deformity would shorten the correction time and reduce the patient's pain [87]. Conventional Ilizarov external fixator and hexapod fixator are two external fixators used in the management of foot and ankle deformities [86].

It is reported in a study that hexapod fixators provide the ability to accurately adjust the bone fragment position in all 3 axes by adjusting just the length of 6 interconnecting struts. A variety of clinical indications, including fracture management, deformity correction, limb lengthening, and joint arthrodesis can be managed using this fixator. The ability to simultaneously correct complex deformities make hexapod frames especially attractive to manage foot and ankle deformities [88]. These procedures enable corrections that are more anatomical, for different degrees of severity and stiffness of deformity [89].

A study conducted in Brazil correcting neglected clubfoot deformity using hexapod fixators concluded that treatment of neglected clubfoot using an external fixator has a high rate of good and excellent results, with a low frequency of complications [89]. The high costs of this method is one of the disadvantages reported in the literature. However, the increased use of this technique would result in money-saving considering its benefits [90].

### Impact of COVID-19 on fracture management

COVID-19 pandemic has placed tremendous pressure on the health care system because of the high prevalence of COVID-19, limited resources and staff, increased risks of transmission, and burden on the health system during the pandemic [22, 83]. In many countries, elective surgeries are cancelled whilst trauma and emergency services are being considered during a pandemic [22]. It is predicted that once the pandemic condition is under control, there will be a surge for orthopaedic surgeries because of restrictions imposed during the pandemic. Many trauma conditions have been managed conservatively along with traditional established orthopaedic principles and require a second stage corrective procedure [22].

A study was conducted in India to compare open fracture incidences and other associated factors related to fractures before and during the pandemic; according to this study, roadside accidents were predominant (73.07%, n = 30) cause for open fractures, the tibia was the common bone involved (23.72%) and EFs were commonly used to manage patients (71.18%, n = 42) with open fracture during the pandemic [91]. In this study, it was observed that there is a delay in presentation to the emergency room and increased use of EFs as a temporary fixation [91]. A study conducted in Iran indicates that the number of pediatric patients attending with pediatric trauma has declined with COVID 19 pandemic [92]. In Sri Lanka, Lockdown resulted in fewer than normal patient loads resulting from road traffic accidents as was the experience of the authors while conducting a patient study on an experimental fixator, which may have further compounded by fuel shortages, due to an ailing economy, although no study has been conducted to verify these observations with data.

Another study analysed the challenges and considerations to resume orthopaedic surgeries during post-COVID-19. A deficient supply of surgical materials such as masks, gloves, and Personal Protective Equipment, operation theatre consumables, orthopaedic implants and instruments, increased expenses to the patients following elaborate protocols during surgeries, and availability of suitable operating theatres are possible challenges [22]. Availability of required surgical materials due to international and national travel restrictions, the possibility of price hikes of fixators due to more demand and less supply are a few factors to consider in orthopaedic surgeries, especially in developing countries, as experienced by the authors while conducting a clinical study on external fixators in Sri Lanka, during the pandemic.

### Need for local manufacturing of EFs

Many developing countries face increased challenges in the healthcare system due to a weakening economy. COVID-19 has accelerated this scenario as there is a need for additional spending to manage COVID-19 related health issues. The increased strain on the healthcare system directly impacts the quality of health services provided for the general public. Existing problems such as war, poor health, and safety measures in developing countries cause more trauma incidences every day. This results in many fractures, requiring external fixation and, in some cases, amputations. Many developing countries rely on imported medical devices for fracture management. These are imported by paying high costs, which place an additional burden on the health care system in developing countries. However, many research studies have shown that locally manufactured EFs are effective in managing fractures. Therefore, it is high time to initiate appropriate measures to manufacture clinically acceptable, low-cost, locally manufactured EFs in developing countries to minimise the dependency on imported devices and the burden on the healthcare system to a certain extent.

Manufacturing essential medical devices locally should be initiated. However, many factors should be considered before initiating this process. Local production and access are associated with each other. Local production is when production takes place in-country to produce biomedical products, and access includes lower prices with greater affordability and greater availability through the presence of locally made products and local distribution networks [3]. A potential benefit of local production is low cost. However, the cost-saving depends on the type of product manufactured and the processing steps. Improving the security of supply and extending procurement options are other advantages of local production. Regular monitoring of the quality of the product by local health authorities will provide improved quality standards without compromising on cost and local production likely to offset the large import deficit and foreign exchange exposure [3]. The development of human capital is another potential benefit of local production [3]. Collaboration within academic institutions and experienced local professionals with knowledge of manufacturing within an industrial environment are likely to occur with local production.

It is important to perform a comprehensive analysis of how local production will affect the price, including the cost for material import, cost related to fabrication and cost associated with biomechanical testing facilities, analyses on accessibility, affordability, and public health needs for EFs. Further, policy and regulations favouring local manufacture with tax credits for local manufacturers and a buy-back guarantee from the government and other factors should be considered before manufacturing EFs locally.

## Summary

In developing countries, EFs have a wide variety of orthopaedic applications. This review article summarised the importance of EFs in developing countries in managing fractures, the problems encountered during external fixation by developing countries, solutions used to address these issues, and the use of external fixation into other domains of treatment.

Although EFs are very expensive, research has been conducted to overcome these barriers in developing countries. However, there are limitations such as the efficacy and safety of low-cost EFs that should be analysed before it is deployed in clinical settings. Further, EFs can be reused several times, and the safety of long-term application and subsequent reuse of reused EFs should be studied.

In developing countries, EFs are widely used for reconstructive surgeries such as deformity correction because of neglected conditions, management of neglected trauma, which leads to deformities, and loss of limb length. It is important to have affordable and clinically acceptable EFs.

## Data Availability

Not applicable.
